# Update on checkpoint blockade therapy for lymphoma

**DOI:** 10.1186/s40425-015-0079-8

**Published:** 2015-07-21

**Authors:** Justin Kline, Michael R. Bishop

**Affiliations:** Department of Medicine, University of Chicago, 5841 S. Maryland Ave., MC2115, Chicago, IL 60637 USA; Committee on Immunology, University of Chicago, Chicago, IL USA

## Abstract

Although cancer cells express antigens recognizable to the immune system, tumors employ a number of diverse mechanisms aimed at subverting the host anti-tumor immune response. Tumor immune evasion pathways have been most thoroughly studied in solid tumors. However, emerging data has demonstrated that malignancies of hematopoietic origin are also able to co-opt their local environment in order to escape immune attack. Activated T cells upregulate negative costimulatory receptors, such as programmed death-1 (PD-1) and cytotoxic lymphocyte antigen-4 (CTLA-4). Engagement of PD-1 or CTLA-4 with ligands expressed on tumor cells or professional antigen presenting cells results in down-regulation of effector T cell function and represents a potent mechanism of immune evasion across a number of human cancers. Antibodies which block PD-1 / PD-L1 interactions have demonstrated remarkable activity in a number of solid tumor subtypes. Interestingly, recent data have demonstrated that in select subtypes of Hodgkin (HL) and non-Hodgkin lymphoma (NHL), the PD-1 ligands are over-expressed due to a genetic amplification of the loci encoding them. Other mechanisms of PD-L1 over-expression in lymphoma have also been elucidated. Reports from early-phase clinical trials of PD-1 blockade have demonstrated remarkable effectiveness in HL, and also appear active against some NHLs. We review the mechanisms of PD-L1 expression in lymphoma and also the early results of anti-PD-1 therapy in this disease.

## Background

Although host immune responses can be spontaneously generated against malignant cells, the tumor environment employs pathways which effectively restrain the expansion and function of invading immune cells. Recently, the disabling of tumor immune evasion mechanisms has led to major breakthroughs in the cancer immunotherapy field. In particular, the administration of antibodies which interrupt interactions between negative regulatory receptors on tumor-specific T cells and their ligands on tumor cells or antigen-presenting cells (APCs) has resulted in impressive clinical activity in a number of human cancers [1]. The PD-1 / PD-L1 (programmed death-ligand 1) pathway has emerged as a highly relevant immune checkpoint pathway in a number of solid tumor types. For example, overall response rates to anti-PD-1 therapy are approximately 30–40 % in melanoma [2], and are quite durable in a fraction of responding patients. The clinical effectiveness of PD-1-blocking antibodies has correlated with PD-L1 expression on tumor or stromal cells in some, but not all patients. Biomarkers capable of identifying patients more likely to benefit from anti-PD-1 and other checkpoint blockade therapies are actively being sought. Recently, recurring genetic amplifications of the loci encoding the PD-1 ligands have been discovered in a subtype of classical Hodgkin lymphoma (HL), and in a genetically related non-Hodgkin lymphoma (NHL), indicating that PD-L1 and/or PD-L2 over-expression on lymphoma cells may play a critical role in immune evasion by these cancers [3]. Recent studies of PD-1 blockade in lymphomas have reported impressive response rates and have generated significant excitement for the further development of new and effective immunotherapies for these diseases [4, 5].

### Mechanisms of increased PD-L1 expression in lymphoma

Classically, expression of PD-L1 on tumor cells is regulated by IFN-γ production, typically by T cells in the tumor environment [6]. In several lymphoma subtypes, including classical nodular sclerosing Hodgkin lymphoma (CNSHL) and mediastinal large B cell lymphoma (MLBCL), a genetic basis for PD-L1 expression in malignant B cells has been uncovered [3]. Here, frequent amplifications of chromosome 9p24.1 have been detected. Key loci at the 9p24.1 amplification peak included *PD-L1, PD-L2* and Janus kinase 2 (*JAK2*). The increased copy number of *PD-L1* and *PD-L2* in affected lymphoma cells correlated with increased protein expression, which was further amplified by dysregulated Jak2 signaling. In a subsequent study of tumor samples from relapsed/refractory CNSHL patients enrolled onto a clinical trial of PD-1 blockade, all 10 patient samples analyzed harbored increased copy numbers of *PD-L1* and *PD-L2*, implicating genetic amplification of the PD-1 ligands in Hodgkin/Reed-Sternberg (HRS) cells as a common mechanism of immune evasion by CNSHL, particularly as the disease progresses. Interestingly, 9p24.1 amplifications have not been identified in mixed cellularity Hodgkin lymphomas (MCHL) [3], which also frequently over-express PD-L1 [7]. In the case of MCHL and other EBV-associated lymphomas, PD-L1 expression is driven by activator protein-1 (AP-1) signaling or possibly through an EBV LMP-1-induced mechanism [7]. In contrast, PD-L1 expression on other lymphomas, where analyzed, appears to occur more sporadically [8, 9]. These elegant observations provided strong rationale for clinical translation of checkpoint blockade therapy with anti-PD-1 antibodies in selected Hodgkin and non-Hodgkin lymphomas.

### Clinical trials of PD-1 blockade in lymphoma

Based on these pre-clinical observations, as well as the efficacy of anti-PD-1 therapy in solid cancers, two early-phase clinical trials of anti-PD-1 antibodies have been conducted in patients with hematological cancers [4, 5]. In both, HL patients were enrolled on independent expansion cohorts, and the results in these patients have recently been reported. The first study of the anti-PD-1-blocking antibody, nivolumab, enrolled 23 patients with relapsed/refractory HL (22 with CNSHL) [4]. Patients were treated at a dose of 3 mg/kg every 2 weeks until achievement of a complete response (CR), or until the point of tumor progression or development of intolerable side-effects. Approximately 80 % had progressed after either autologous stem cell transplantation or following therapy with brentuximab vedotin. The overall response rate (ORR) was 87 % with a CR rate of 17 %. Eighty-six percent of patients were progression-free at 24 weeks. Several responding patients went on to receive an allogeneic stem cell transplant. The therapy was overall well-tolerated with few grade 3 or 4 toxicities reported. Although longer follow-up is warranted to document duration of responses, the ORR following PD-1 blockade in HL exceed those observed in solid cancers. One caveat is that all but one of the HL patients on this study had CNSHL, so the effectiveness of PD-1 blockade in other HL subtypes remains to be proven. The second report, published in abstract form, included 15 HL patients treated with another PD-1-blocking antibody, pembrolizumab, at a dose of 10 mg/kg every 2 weeks until disease progression or the development of excessive toxicity [5]. All patients had progressed after brentuximab vedotin, and two-thirds had previously received an autologous stem cell transplant. The ORR was 53 %, with a CR rate of 20 %. Here again, pembrolizumab therapy was well tolerated, with only 1 grade 3 toxicity reported.

There are also emerging data suggesting that checkpoint blockade therapy (CBT) with PD-1-blocking antibodies may be effective in NHL. Investigators at the M.D. Anderson Cancer Center recently published their results of a phase II study of a third PD-1-blocking antibody, pidilizumab, in combination with the anti-CD20 antibody, rituximab, for patients with relapsed follicular lymphoma [10]. This combination was well tolerated and induced an ORR and CR rate of 66 and 52 %, respectively. Although it is impossible to tease out the direct effect of PD-1 blockade in this study, the ORR to the combination appeared promising compared to historical controls treated with rituximab alone. Lastly, the preliminary results of the phase I study of nivolumab in patients with refractory hematological malignancies were recently reported [11]. Patients with diffuse large B cell lymphoma (*n* = 11) and follicular lymphoma (*n* = 10) achieved a 36 and 40 % ORR, respectively. Clearly, PD-1 blockade therapy is active in NHL. However, the follow-up duration on this study is quite short.

## Conclusions

The unique genetic mechanisms underlying PD-L1 over-expression in CNSHL and PMBL are fascinating and indicate that, in order to subvert an ongoing anti-tumor immune response, these lymphomas activate PD-1 ligand expression [3]. PD-L1 expression also occurs in other lymphoma subtypes, but less frequently [8, 9]. Early results of CBT with anti-PD-1 antibodies have revealed that they are safe and associated with extremely high response rates in HL [4, 5]. The FDA has recognized the promise of CBT in HL, and granted nivolumab Breakthrough Therapy Designation for the treatment of patients with HL after failure of autologous stem cell transplant and brentuximab vedotin. Larger studies of PD-1-blocking antibodies in HL are underway. It is interesting to begin to conceptualize how these agents will ultimately be incorporated in the treatment of HL patients, and whether manipulation of other immune checkpoints in HL will be effective. For this reason, it will be important to define whether HRS cells commonly express additional inhibitory ligands that could be targeted therapeutically. It will also be crucial to identify predictive biomarkers of response to CBT in HL and NHL. For example, does mutational burden in lymphoma correlate with responsiveness to CBT as recently demonstrated in melanoma and lung cancer [12, 13]? Lastly, the antigens expressed on lymphoma cells that are recognized by T cells in the microenvironment are largely unknown. Neo-antigen discovery through exome and RNA sequencing, combined with HLA binding prediction and validation, represents a major next step in improving our understanding the mechanisms which underscore responsiveness to CBT therapy in lymphoma. Although the numbers of treated patients are small and follow-up duration limited, the PD-1 story in HL is one that highlights the importance of basic scientific discoveries paving the way for translation of effective cancer therapies into the clinic.
